# Retrospective single-center study of endogenous endophthalmitis, Northeastern China, 2010-2023

**DOI:** 10.3389/fcimb.2026.1805346

**Published:** 2026-09-03

**Authors:** Deng Zhang, Feng Gu, Xiaoyu Zhang, Xifan Zhang, Sufei Tian, Baiyi Chen, Yunzhuo Chu, Jingping Zhang, Xin Zhang

**Affiliations:** 1 Department of Infectious Disease, Xiamen Xinglin Hospital, Xiamen, China; 2 Department of Ophthalmology,The First Hospital of China Medical University, Shenyang, China; 3 General Intensive Care Unit, Zhaoqing Hospital, The Third Affiliated Hospital of Sun Yat-sen University, Zhaoqing, China; 4 First Department of Infectious Disease, The First Hospital of China Medical University, Shenyang, China; 5 Department of Laboratory Medicine, The First Hospital of China Medical University, Shenyang, China

**Keywords:** endogenous endophthalmitis, high-pathogenicity *Klebsiella pneumoniae*, prognosis, treatment, virulence factors, whole genome sequencing

## Abstract

**Objective:**

To explore the epidemiological characteristics of EnE and the molecular traits of the *Klebsiella pneumoniae*(KP).

**Methods:**

We revived 40 strains of the KP, utilized whole-genome sequencing technology to understand their molecular epidemiological characteristics.

**Results:**

Among 103 EnE patients (69.90% male, 56.31% left eye), vision loss was the chief complaint (96.12%). Extraocular infections were present in 63.11% of patients, most commonly liver abscess (45.00%). KP comprised 81.82% of culture-positive cases. Among 40 KP strains analyzed, 82.5% were hypervirulent KP (hvKP). Phylogenetic analysis revealed the dominance of ST23-K1 lineages. Intravitreal injection was associated with a significantly reduced risk of poor visual prognosis (RR = 0.061, 95%CI: 0.005–0.442) and emerged as an independent protective factor.

**Conclusions:**

The incidence of EnE showed an increasing trend, primarily driven by hvKP ST23-K1. A comprehensive search for systemic infection foci, particularly liver abscesses, is essential. A negative string test result does not exclude hvKP. Timely intravitreal antimicrobial injection is a critical modifiable factor for improving visual prognosis.

## Highlights

- For patients with Klebsiella pneumoniae bacteremia or liver abscesses, clinicians should inquire about ocular symptoms and perform ophthalmic examinations.
- A negative string test cannot exclude hypervirulent strains.
- Intravitreal antibiotic injection contributes to improved visual prognosis.

## 1 Introduction

Endogenous endophthalmitis (EnE) is a metastatic infection caused by pathogens disseminating via the bloodstream to the eye (Gurnani and Kaur, 2023), typically with poor prognosis and prone to clinical misdiagnosis or missed diagnosis (Sadiq et al., 2015). In recent years, the incidence of EnE caused by Gram-negative bacteria, particularly *Klebsiella pneumoniae* (KP), has gradually increased, becoming an important type of EnE in East Asia (Chen et al., 2025). *Klebsiella pneumoniae*-related endogenous endophthalmitis (EKE) is frequently complicated with distant invasive lesions such as liver abscesses (Lee et al., 2012; Lee et al., 2024), and its poor prognosis is largely associated with the hypervirulent features of strains (Chung et al., 2017). Whole−genome sequencing (WGS) enables comprehensive analysis of the genetic background and virulence characteristics of such strains (Brisse et al., 2013; Jh et al., 2019; Wei et al., 2021; Tan et al., 2024; Fernández Vecilla et al., 2025; Lam et al., 2025).

Among current studies on endogenous endophthalmitis (EnE), the proportion of EnE among all endophthalmitis cases varies widely (3%–19%) across reports due to geographical, genetic and methodological differences (Deng et al., 2025). Moreover, existing studies mostly focus on general population-based analyses, while integrated clinical-molecular investigations specifically for EKE remain scarce. The clinical differences between EKE and non-EKE cases, the prognostic value of ocular invasive therapy, and risk factors for adverse outcomes have not been systematically clarified. Therefore, this research conducts multi-dimensional analyses covering epidemiology, clinical features, molecular phenotypes of isolates, phylogeny, intervention strategies and prognostic risk factors, to characterize the specific clinical-molecular signatures of EKE. It aims to provide evidence for the clinical diagnosis, treatment and prognostic evaluation of EnE, especially EKE.

## 2 Research objects and methods

### 2.1 Research objects

The medical records of inpatients diagnosed with endophthalmitis in a tertiary hospital in Northeast China from June 2010 to June 2023 were collected through the electronic medical record system, which were consecutive cases, and only the first hospitalization was counted for multiple hospitalizations of the same patient. All laboratory findings were referenced to the initial laboratory tests at the time of admission. This research is a retrospective study, and the cases were included without obtaining informed consent forms. This research received ethical approval from the Institutional Research Ethics Committee, with approval number 2023-535-2.

1. Inclusion criteria: ① Hospitalized patients clinically diagnosed with endogenous endophthalmitis (EnE) between June 2010 and June 2023;② Complete clinical data available.
2. Exclusion criteria: ① Cases of exogenous endophthalmitis, including post-operative endophthalmitis (defined as occurring within 1 year after ophthalmic surgery), traumatic endophthalmitis, endophthalmitis associated with glaucoma filtering surgery, and keratitis-induced endophthalmitis;② Uveitis caused by viruses, parasites and other pathogens;③ Intraocular inflammation induced by other etiologies (e.g., non-infectious uveitis secondary to tumor necrosis or intraocular foreign bodies).

### 2.2 Collection of general information

The clinical symptoms and related laboratory indicators of the patients were the manifestations at the time of their admission and the initial test results obtained.

### 2.3 Drug susceptibility test and string test

The 40 KP strains included in the study were analyzed using the French Bio-Mérieux VITEK 2 automatic bacterial identification and drug susceptibility analysis system, and the drug sensitivity test results were determined to be sensitive, mediated, and resistant according to the American Institute of Clinical and Laboratory Standards standards (CLSI, 2023). Extended-spectrum beta-lactamase(ESBL) strains were detected by double-sheet synergy test and phenotypic confirmation test.

The strain was inoculated in blood agar medium by three-stage scribing method, and after 18~24 hours of culture in an incubator at 35 °C, a single colony was dipped in a bacterial inoculation ring to pick up the colony. If the length of the mucus filament of the provoked colony was ≥ 0.5 cm, it was a positive strain in the string test, and another experimenter repeated the test to confirm.

### 2.4 whole genome sequencing

The 40 EKE strains were sequenced in high-throughput format by the Nanjing Paseyno Biotech Co., Ltd. using the WGS sequencing strategy to construct a library of different insertion fragments. The second generation sequencing technology was used to sequence the library in paired-end format based on the IlluminaNovaSeq sequencing platform.

### 2.5 Data quality control and filtering

The quality control of the sequencing data is performed by fastp software, and the raw data is filtered to generate high-quality sequence. The standards are as follows: ① removal of connector contamination; ② length filtering: if the length of any of the ends of the reads is ≤50bp, the ends of the reads are removed; ③ reads quality filtering: if the average quality value of any of the ends of the reads is<20, the ends of the reads are removed; ④ fuzzy base N filtering: if the number of N bases in any of the ends of the reads is ≥5, the ends of the reads are removed. The basic situation of data filtering is shown in **Annexed Table 1**.

### 2.6 Genome assembly and effect evaluation

The data of the second generation were assembled from scratch using the SPAdes software, and the scaffolds with an average depth of more than 10 and a length of more than 500 bp were selected as the assembly results, and the scaffolds and contigs were constructed. The assembly results were obtained by correcting the single base using the Pilon software, and the assembly results are shown in **Annexed Table 2**.

### 2.7 Public database KP genome information acquisition

To conduct a background comparative analysis of virulence and drug-resistance genes, 31 complete genome sequences of KP were selected from the NCBI GenBank database via convenience sampling. The retrieval and screening criteria were as follows: species limited to Klebsiella pneumoniae, collection period from 2004 to 2025, global geographical origin, all isolated from human clinical specimens, with environmental and animal-derived strains excluded. The isolation sites included blood, pus, liver abscesses, ocular secretions, etc. These 31 reference strains were not precisely matched with the endogenous endophthalmitis-causing KP strains in this study with respect to sequence typing, serotyping or geographical origin, and were only used for background comparative research at the genetic level.

### 2.8 Genetic analysis

The full genome data of 71 KP were uploaded to the global genomic monitoring platform https://pathogen.watch/ (Argimón et al., 2021) and the distribution of ST typing, serotype, iron carrier and a series of virulence-related regulatory genes was obtained.

The K.pneumoniae (KP-Z, SAMN59993439 https://www.ncbi.nlm.nih.gov/biosample/59993439) strain was selected as the reference genome for the SNP analysis. The strain was isolated in 2021 from a patient in our hospital with both a liver abscess and a lung abscess, and was a hvKP (ST23, K1, *wzi*1, *iucA* (+), *iroB* (+), *rmpA* (+), *rmpA2* (+), *peg-344* (+)). The phylogenetic tree was constructed using the neighbor-joining method based on the SNP results and was visualized on the Chiplot website (https://www.chiplot.online/).

### 2.9 Definition criteria

1. Operational Definition of EnE: EnE refers to intraocular infection caused by hematogenous pathogen spread without exogenous triggers such as ocular trauma or ophthalmic surgery.①Clinical criteria: Acute intraocular inflammation with systemic infection manifestations or distant infectious lesions. ②Microbiological criteria: Positive culture of aqueous humor or vitreous samples; culture-negative cases were diagnosed by clinical and systemic evidence.
2. Definition criteria for hypervirulent In this research, the strains of *peg-344, iroB, iucA, rmpA*, and *rmpA2* coexisting as hvKP were shown in the WGS results.
3. Definition of Outcome Measures: The primary outcome measure of this study was poor visual prognosis, which was defined as final visual acuity of no light perception, light perception, or finger counting at follow-up, or performance of eyeball enucleation/evisceration due to uncontrolled infection and severe destruction of ocular structures. Best-corrected visual acuity ≥0.02 was defined as good visual prognosis.

### 2.10 Statistical analysis

The data were processed using SPSS 27.0 software. For normally distributed measurement data, the t-test was used, and for non-normally distributed measurement data, the Mann-Whitney U test was used. χ^2^ test was performed on qualitative data, with *P* < 0.05 indicating statistical significance. Factors with *P* < 0.1 were included in the logistic regression model, with *P* < 0.05 indicating statistical significance.

## 3 Results

### 3.1 Epidemiological characteristics of EnE

**Figure 1:**
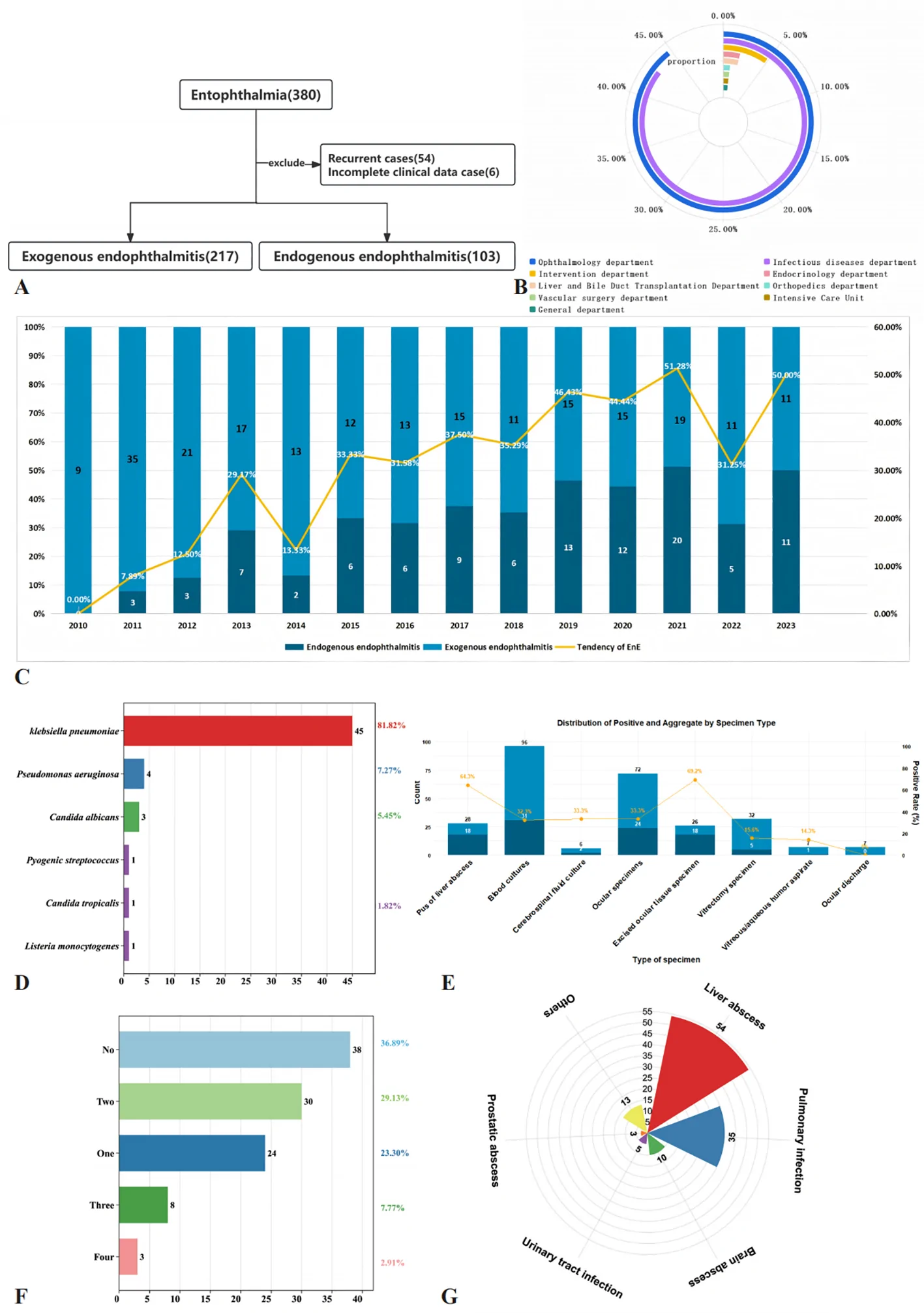
Process of case selection and epidemiological characteristics of EnE. **(A)** A total of 380 cases clinically diagnosed with endophthalmitis were identified, with 54 duplicate cases and 6 cases with incomplete clinical data excluded, leaving a total of 320 cases, of which 217 were exogenous endophthalmitis (67.81%) and 103 were endogenous endophthalmitis (EnE) (32.19%). **(B)** Departmental distribution of EnE cases. **(C)** Trend of EnE case numbers from 2010 to 2023. Due to the limited project duration, only data from January to June 2023 were included, representing an incomplete half−year dataset. **(D)** Pathogen composition of EnE, in descending order: Klebsiella pneumoniae (81.82%), Pseudomonas aeruginosa (7.27%), Candida albicans (5.45%), Streptococcus pyogenes (1.82%), Candida tropicalis (1.82%), and Listeria monocytogenes (1.82%). **(E)** Culture positivity rates of different types of specimens. **(F)** Number of extraocular invasive infection foci. Cases with 2 infectious foci accounted for 29.13% (30 cases), followed by those with 1 focus (23.30%, 24 cases), 3 foci (7.77%, 8 cases), and 4 foci (2.91%, 3 cases). **(G)** Distribution of extraocular invasive infection foci. Liver abscess was the most common extraocular infectious focus, accounting for 45.00% (54 cases), followed by pulmonary infection (29.17%, 35 cases), brain abscess (8.00%, 10 cases), urinary tract infection (4.00%, 5 cases), prostatic abscess (3.00%, 3 cases), and infections at other sites (11.00%, 13 cases).

A total of 320 cases of endophthalmitis were included in this research, including 217 cases (67.81%) of exogenous endophthalmitis and 103 cases (32.19%) of EnE, and the screening process is detailed in **Figure 1A**. Of the 103 hospitalized patients with EnE, 44% were in ophthalmology and 41% were in infectious diseases, as detailed in **Figure 1B**. From 2010 to 2023, the number of EnE cases showed an overall upward trend. Since 2018, the proportion of EnE has exceeded 40% multiple times, particularly in 2021 and 2023, where it was ≥50%, while 2022 saw a slight decrease (31.25%). The trend of annual changes in the number of hospitalized patients with EnE is shown in **Figure 1C**.

Among the 103 EnE cases, 55 were cultured positive, of which 45 were KP (81.82%), and the pathogen distribution is shown in **Figure 1D**. The positive rate of culture of resected eye tissue specimens was the highest, reaching 69.23% (18/26), the positive rate of liver abscess puncture and drainage was also very high, 64.29% (18/28), and the positive rate of blood culture was 32.29% (31/96), and the positive rate of various specimens is shown in **Figure 1E**. The proportion of extracranial co-infection foci was as high as 63.11%. The cases with two infection foci accounted for the most (29.13%), followed by one infection focus (23.30%), three infection foci (7.77%), and four infection foci (2.91%), as shown in **Figure 1F**. Liver abscess was the most common extracranial lesion (45.00%), followed by pulmonary infection (29.17%), as shown in **Figure 1G**. Specific values are presented in the **Annexed Table 3**.

### 3.2 Clinical characteristics of patients with EnE

#### 3.2.1 The overall clinical characteristics of patients with EnE

Among the 103 cases of EnE, there were more males (69.90%) than females (30.10%), with an average age of 59.6 ± 11.964 years. In 56.31% of cases, the left eye was affected, and approximately 9.71% of cases involved both eyes simultaneously. Diabetes was the most common underlying disease, and vision loss was the most common initial symptom. See **Table 1** for details.

**Table 1:** Basic characteristics of research objects.

Variate		Proportion
Sex	Male	72(69.90%)
	Female	31(30.10%)
Age, years		59.60 ± 11.964
Length of hospital stay, days		20(10, 34)
Affected only one eye	right eye	35(33.98%)
	left eye	58(56.31%)
Affected both eyes		10(9.71%)
Combined diabetes, n (%)		61(59.22%)
Combined hypertension, n (%)		27(26.21%)
Recent surgical history		8(7.77%)
Inserted catheter		5(4.85%)
History of drug abuse		1(0.97%)
History of smoking		25(24.27%)
History of drinking		20(19.42%)
Initial BCVA logMAR		2.903(2.602, 3.204)
Final BCVA logMAR		3.204(2.602, 3.204)
Initial symptoms	Diminution of vision	99(96.12%)
	Symptoms of eye pain	63(61.17%)
	Red eyes	49(47.57%)
	Fever	67(65.05%)
	Chills	41(39.81%)

BCVA, Best corrected visual acuity; Recent surgical history does not include ocular surgeries.

#### 3.2.2 Comparative analysis of EKE and non-EKE clinical data

We divided 103 EnE patients into EKE group and non-EKE group according to whether KP was cultured, with the results detailed in **Tables 2**–**4**. The white blood cell count, neutrophil ratio, liver enzyme, and bilirubin levels in the EKE group were higher than those in the non-EKE group, while the platelet count and albumin levels were relatively lower, and fever symptoms were more common in the EKE group.

**Table 2:** Comparison of differences in clinical laboratory indicators between the EKE group and the non-EKE group.

Indicators	Groups	$\bar{x}\pm \text{s}$	P Value	Mean range	95%CI
HGB	EKE(n=45)	119.29 ± 21.81	0.074	7.97	(-7.87, 16.73)
	Non-EKE(n=58)	127.26 ± 22.71			
ALB	EKE(n=45)	29.19 ± 5.03	<0.001	4.49	(1.89,7.10)
	Non-EKE(n=58)	33.69 ± 7.61			

HGB (hemoglobin) and ALB (serum albumin) follow a normal distribution. An independent sample t-test was conducted. P<0.05 indicates that the difference is statistically significant.

**Table 3:** Comparison of clinical laboratory indicators between EKE group and non-EKE group.

Indicators	Median of the EKE group (n=45)	Median of the non-EKE group (n=58)	U	P value
WBC(×109/L)	13.21	9.75	784.00	<0.001
NE(×109/L)	11.09	7.47	726.50	<0.001
NE%(%)	85.00	76.85	717.50	<0.001
PLT(×109/L)	177.00	242.00	946.50	0.017
ALT(U/L)	42.00	26.50	933.00	0.013
ALP(U/L)	143.00	110.00	786.50	<0.001
GGT(U/L)	120.00	60.00	822.00	0.001
TBIL(umol/L)	20.00	12.10	685.00	<0.001

WBC (white blood cell count), NE (neutrophil count), PLT (platelet count), ALT (alanine aminotransferase), ALP (alkaline phosphatase), GGT (γ-glutamyl transferase), TBIL (total bilirubin). These clinical indicators do not follow a normal distribution. The Mann-Whitney U test was conducted, and P<0.05 indicates a statistically significant difference.

**Table 4:** Differences in clinical symptoms between the EKE group and the non-EKE group.

Indicators		EKE (n=45)	Non-EKE (n=58)	Total (n=103)	χ^2^	P value
Fever	Yes	35(77.8%)	32(55.2%)	67(65.0%)	5.695	0.017
	No	10(22.2%)	26(44.8%)	36(35.0%)		
Chills	Yes	22(48.9%)	19(32.9%)	41(39.8%)	2.752	0.097
	No	23(51.1%)	39(67.2%)	62(60.2%)		
Fear of cold	Yes	11(24.4%)	8(13.8%)	19(18.4%)	1.911	0.167
	No	34(75.6%)	50(86.2%)	84(81.6%)		
Affected both eyes	Yes	4(8.9%)	6(10.3%)	10(9.7%)	0.062	0.804
	No	41(91.1%)	52(89.7%)	93(90.3%)		
Diminution of vision	Yes	44(97.8%)	55(94.8%)	99(96.1%)	~	0.41
	No	1(2.2%)	3(5.2%)	4(3.9%)		
Symptoms of eye pain	Yes	27(60.0%)	36(62.1%)	63(61.2%)	0.046	0.831
	No	18(40.0%)	22(37.9%)	40(38.8%)		
Red eyes	Yes	26(57.8%)	33(56.9%)	59(57.3%)	0.008	0.929
	No	19(42.7%)	25(43.1%)	44(42.7%)		

①The statistical data of clinical symptoms are qualitative data and are analyzed using the chi-square test; ②When≥2 minimum theoretical frequencies 1≤T<5, the Fisher exact probability method is used. P<0.05 indicates that the difference is statistically significant.

### 3.3 The microbiological characteristics of EKE

#### 3.3.1 Antibiotic Susceptibility, ST Typing, Capsular Serotyping, and wzi typing results

**Figure 2:**
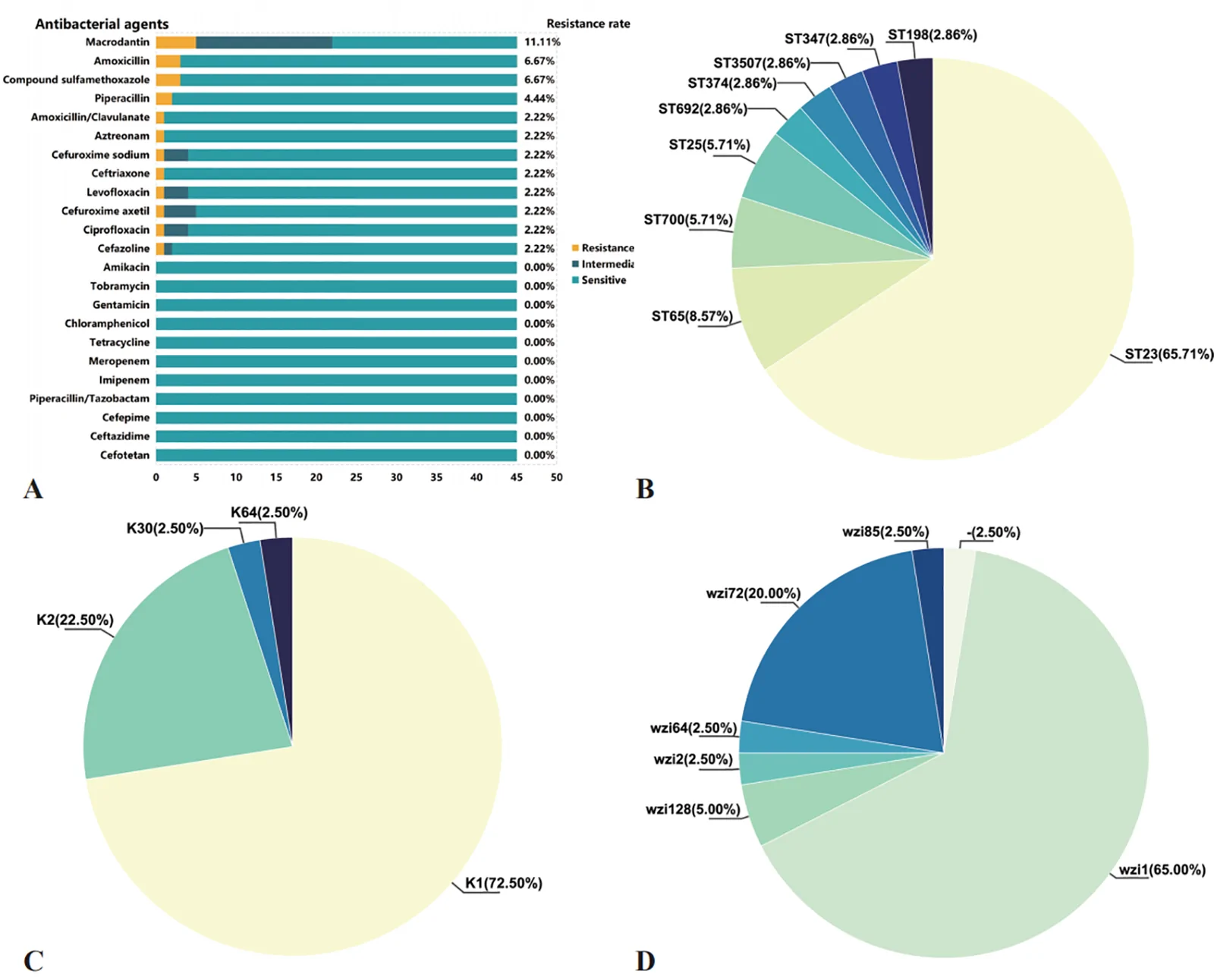
Antibiotic susceptibility results and ST classification, capsule serotype, and wzi classification of KP. **(A)** The drug sensitivity results of KP, arranged from the highest to the lowest resistance rate of KP to the drugs. **(B)** The distribution of ST types. **(C)** The distribution of capsular serotypes. **(D)** The distribution of wzi types.

Among the 45 EKE patients, KP specimens of 40 patients were successfully revived. Only one strain was ESBL-resistant, while the rest were sensitive strains, as detailed in **Figure 2A**. MLST typing revealed that ST23 was dominant, accounting for 27 strains (67.5%), while ST65 was found in 5 strains (12.5%). The remaining details are shown in **Figure 2B**. Capsular serotype typing showed that K1 was dominant, accounting for 29 strains (72.5%), while K2 was found in 9 strains (22.5%). Details are shown in **Figure 2C**. *wzi* typing showed that *wzi*1 was dominant, accounting for 26 strains (65%). Details are shown in **Figure 2D**.

#### 3.3.2 Detection of hvKP and related virulence factors and the results of the string test

**Figure 3:**
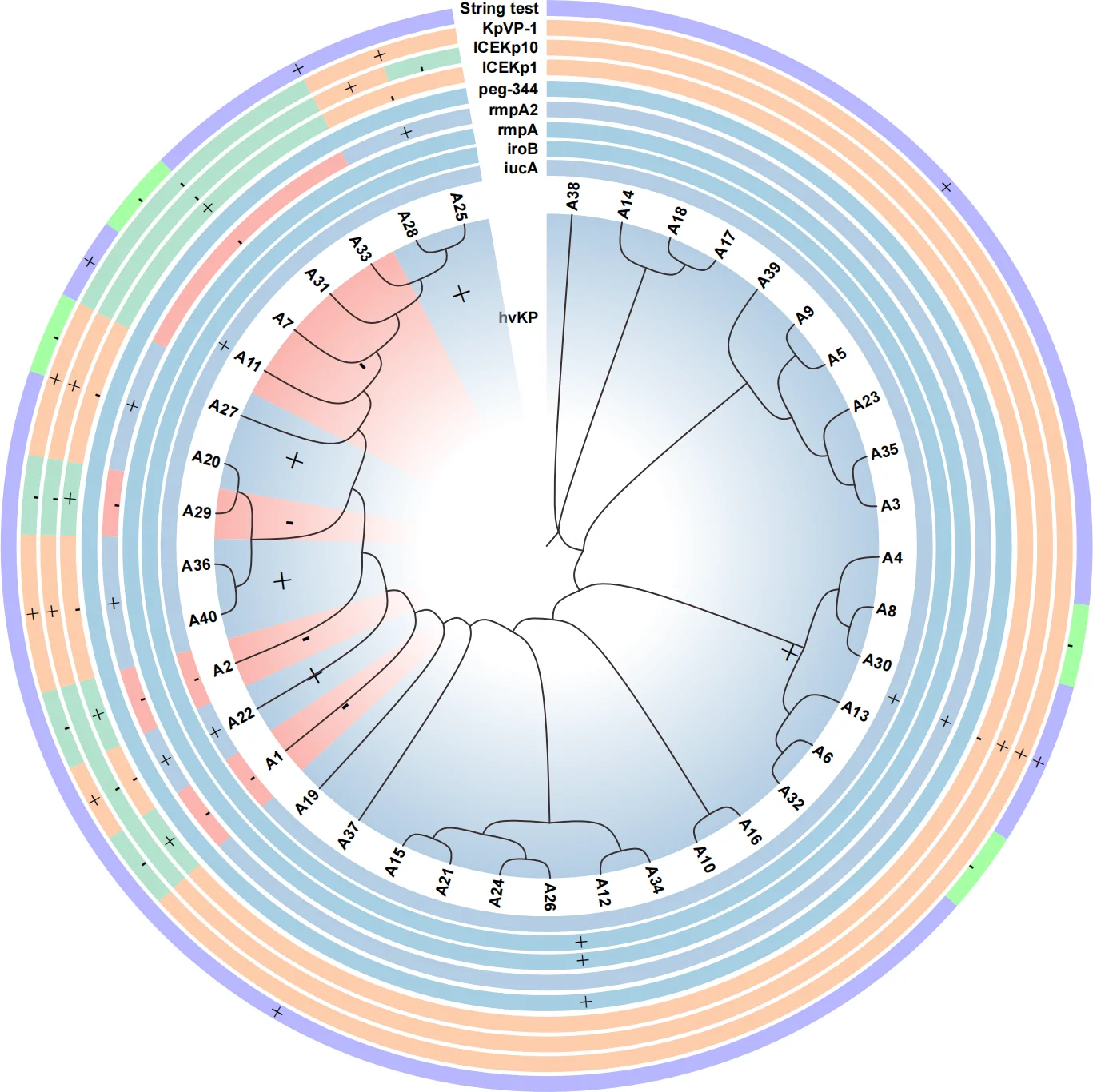
Phylogenetic tree based on SNP, detection of hvKP and related virulence factors, and string test results. The phylogenetic tree of 40 KP plants was plotted based on SNP, and the innermost circle was the detection of hvKP, blue represented hvKP (+), and orange was hvKP (-). The second circle is the specimen number (A1-A40); The distribution of *iucA, iroB, rmpA, rmpA2* and *peg-344* in circles 3~7 was orange and positive. The distribution of ICEKp1, ICEKp10 and KpVP-1 in circles 8~10 was orange and green. The 11th circle is the string test result. Green is the string test (-), and purple is the string test (+).

A total of 33 strains of hvKP were detected, accounting for a high proportion of 82%, with 36 strains (90%) positive in the string test. ICEKp1 was positive in 7 strains (17.5%), and ICEKp10 was positive in 31 strains (77.5%), as detailed in **Table 5**. The correlation between the string test and hvKP is shown in **Figure 3**, with 30 out of 33 hvKP strains positive in the string test (30/33), and 30 out of 36 string test positives being hvKP (30/36).

**Table 5:** Detection of hvKP and related virulence factors and the results of the string test.

Indicators		Number of strains	proportion
The identification combination of hvKP	iucA	38	95.00%
	iroB	40	100.00%
	rmpA	40	100.00%
	rmpA2	33	82.50%
	peg-344	40	100.00%
hvKP		33	82.50%
Other related virulence factors	ICEKp1	7	17.50%
	ICEKp10	31	77.50%
	KpVP-1	33	82.50%
String test		36	90.00%

① iucA encodes the synthetase for aerobactin, a type of iron carrier bacteriocin, and is a key enzyme for the synthesis of aerobactin; ② iroB encodes the synthetase for salmochelin, a type of iron carrier bacteriocin, and is involved in the biosynthesis of salmochelin; ③ rmpA (Regulator of mucoid phenotype A) and rmpA2 (Regulator of mucoid phenotype A2) are both capsule mucin phenotype regulatory factors that can enhance the synthesis of capsule polysaccharides in bacteria and increase virulence; ④ the peg-344 gene is located on the virulence plasmid of hvKp and is one of the specific markers of hvKp strains; ⑤ ICEKp1 and ICEKp10 are important integron-conjugation elements in KP; ⑥ KpVP-1 is one of the high virulence-related virulence plasmid types, carrying multiple key virulence genes.

### 3.4 Phylogenetic analysis of KP strains from public databases and this research

**Figure 4:**
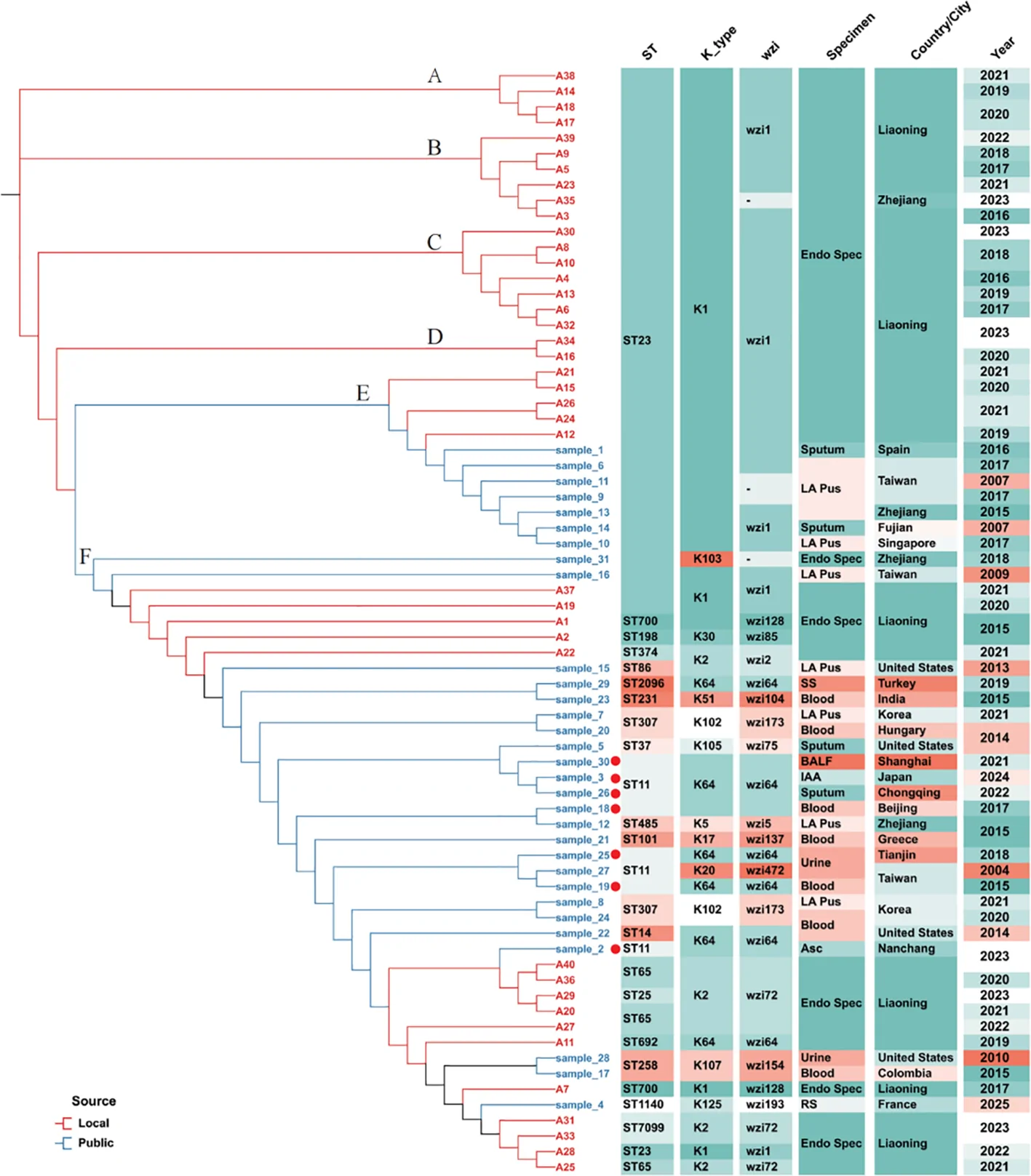
Phylogenetic tree of KP from public databases and this research. The left half is a phylogenetic tree based on SNPs, including 31 KP strains (sample_1~sample_31, blue branch) from public databases and 40 EKE strains (A1~A40, red branch) from the public database. The right part is the information of each matched strain, columns 1~6 are MLST typing, capsular serotype, WZI typing, specimen type, country or city of specimen source, and specimen collection time. Some of the specimen types in column 4 were abbreviated, namely: ASc (ascitic fluid, ascites), BALF (Bronchoalveolar lavage fluid, alveolar lavage fluid), Endo Spec (Endophthalmitis specimen, endophthalmitis specimen), IAA (Intra-abdominal abscess), and LA Pus (Liver abscess pus), RS (Rectal swab), SS (Skin swab). ST11-K64 strains are indicated by red circular markers.

The phylogenetic tree showed that the 71 KP strains were mainly divided into 6 clone groups, each representing a group of strains with a common genetic background. Among them, there were 19 strains in A, B, C, and D branches (all EKE strains). Clade E included a total of 12 strains (4 EKE strains and 8 strains from the public database), including 5 strains of liver abscess specimens (from Taiwan, Zhejiang and Singapore) and 2 sputum specimens (from Spain and Fujian). The strains of the five clades A-E are all ST23-K1 types. The F clade contains the most strains, with 40 strains (16 EKE strains and 24 strains in the public database), the strains of this clade are more diverse, MLST classification includes ST11, ST258, ST65, ST86, ST25, etc., capsular serotypes include K64, K1, K2, etc., ST11 is mostly associated with K64, specimens are derived from blood, urine, sputum, abdominal abscess, skin swab, alveolar lavage, etc., from many provinces in our country and all over the country (United States, South Korea, Colombia, France, etc.), The remaining details are shown in **Figure 4**.

### 3.5 Intraocular invasive interventions

In this research, the number of patients receiving intraocular invasive procedures is shown in **Table 6**. Chi-square analysis revealed that the evisceration rate was significantly lower in patients who underwent pars plana vitrectomy than in those who did not (*P* < 0.05), as presented in **Table 7**.

**Table 6:** Intraocular invasive interventions.

Intraocular invasive interventions	Number of cases	Percentage (%)
Monotherapy		
Intravitreal injection only	21	20.39
Pars plana vitrectomy only	36	34.95
Evisceration only	32	31.07
Combined therapy		
Intravitreal injection + Evisceration	3	2.91
Pars plana vitrectomy + Evisceration	1	0.97
Other multiple combinations	10	9.71
Total	103	100.00

**Table 7:** Comparison of evisceration rates between patients with and without intravitreal injection or pars plana vitrectomy.

		Enucleation of eyeball		χ2	P
		Yes	No		
intravitreal injection	yes	3(12.50%)	21(87.50%)	6.938	0.008
	no	33(41.77%)	46(58.23%)
pars plana vitrectomy	yes	1(2.70%)	36(97.30%)	26.413	<0.001
no	35(53.03%)	31(46.97%)

Among patients who underwent pars plana vitrectomy, 36 had preserved globes, of whom 34 achieved functional visual acuity and 2 were blind at discharge. As this is a retrospective study, long-term post-discharge follow-up data are lacking; therefore, long-term visual prognosis was not evaluated.

### 3.6 Analysis of risk factors associated with poor visual prognosis

A total of 98 patients (95.15%) in this research had poor visual prognosis. The proportion of patients with poor prognosis who received intravitreal injection (79.2%) was lower than that of those who did not receive intravitreal injection (98.7%) RR = 0.061(95%CI:0.005-0.442), as shown in **Table 8**. 95.15% of the patients ultimately had poor visual prognosis (98/103), among whom 36 patients had their eyes enucleated. Poor initial vision was a risk factor for poor visual prognosis. Intravitreal injection could reduce the risk of poor prognosis. The analysis of risk factors for poor visual prognosis is shown in **Table 9**.

**Table 8:** Analysis of the relative risk of poor visual prognosis in subjects receiving intravitreal injection.

		Prognosis of visual acuity		RR	95%CI
		Final	Poor		
Intravitreal injection	No	1(1.3%)	78(98.7%)	0.061	(0.007, 0.495)
Yes	5(20.8%)	19(79.2%)

**Table 9:** Analysis of risk factors for poor visual prognosis.

Factors	Single factor analysis		Multiple factor analysis	
	OR (95%CI)	P Value	OR (95%CI)	P Value
Three extraocular lesions	0.132(0.20-0.871)	0.035		
Left eye affected	6.829(0.769-60.686)	0.085		
Intravitreal injection	0.049(0.005-0.442)	0.007	0.104(0.008-1.310)	0.080
Initial vision	2.576(1.054-6.294)	0.038		
HB	0.961(0.919-1.005)	0.079		
PLT	1.008(1.000-1.017)	0.062		
ALT	0.983(0.966-1.000)	0.048		

ALT: Alanine Aminotransferase - a measure of liver function.

## 4 Discussion

A total of 103 EnE cases were included in this research, accounting for 32.19% of all endophthalmitis cases, which is higher than the 3%~19% mentioned in the previous literature (Deng et al., 2025). Moreover, the proportion has been increasing in recent years, reaching ≥50% in 2021 and 2023. Among culture-positive specimens, 81.82% were KP, which aligns with the global trend of increasing hvKP infections and, consequently, the risk of hematogenous endophthalmitis (Serban et al., 2021). Additionally, advancements in imaging and molecular diagnostic techniques have improved the detection rates of EnE and its occult sources of infection. EnE is no longer a rare disease in clinical practice and should be given importance in the differential diagnosis of endophthalmitis.

For the etiological diagnosis of EnE, higher detection rates are achieved by directly sampling tissue or pus from the infected focus (e.g., resected ocular tissue, liver abscess drainage fluid), while blood cultures identify the most positive cases. EnE primarily relies on clinical diagnosis, with approximately 20% to 30% of endophthalmitis cases remaining negative after standard culturing (Durand, 2017), and reported misdiagnosis rates ranging from 16% to 63% (Maling et al., 2018). When a patient with EnE and liver abscess has indications for puncture, liver abscess drainage should be performed as early as possible, and emerging pathogen detection techniques that offer faster and more sensitive pathogen identification may be used when necessary (Ag et al., 2025).

In this research, the proportion of EnE cases with extraocular infection was as high as 63.11%, of which two extraocular lesions were the most common (29.13%), which is consistent with the conclusions of previous studies (Suo et al., 2025). Among all extraocular lesions, liver abscess accounted for the highest proportion (45.00%), followed by lung infection (29.17%). A meta-analysis published in 2020 summarized 15 retrospective studies worldwide and noted that the pooled incidence of secondary EnE after liver abscess due to KP was 4.5% (95% CI: 2.4%–8.2%) (Hussain et al., 2020). In clinical practice, routine screening of systemic infection foci (especially abdominal and chest imaging) is recommended for patients with EnE with only ocular symptoms to identify the source of infection in a timely manner. In addition, clinicians should actively inquire about and closely monitor ocular symptoms such as visual acuity loss and eye pain for patients who have been diagnosed with liver abscess, especially if the etiology suggests KP infection, and be vigilant against the occurrence of EnE.

The proportion of men (69.90%) was significantly higher than that of women (30.10%) in all EnE cases, but there is currently insufficient evidence to prove that men are more susceptible to EnE, and attention should be paid to observation in future studies. In this research, left eye involvement (56.31%) was more common than right eye involvement (33.98%), while previous literature suggested that the proximal right carotid artery flowed directly to the right eye (Greenwald et al., 1986), and the right eye was theoretically more susceptible, which needs to be confirmed by further research. The analysis of clinical test results showed that fever symptoms were more common in the EKE group, and the white blood cell count, neutrophil ratio, liver enzyme, and bilirubin levels were higher than those in the non-EKE group, while the platelet count and albumin levels were relatively lower, which suggested that in clinical work, if the test of EnE patients showed the above abnormalities and combined with fever, it was easier to obtain positive culture results.

A total of 33 hvKP strains were detected in this research, accounting for as high as 82%. In the string test, four KP strains showed negative results, but three of them exhibited extremely strong invasiveness. These three patients all had liver abscesses, and each was concurrently suffering from pulmonary infection, splenic abscess, and right foot infection. This finding alerts clinicians that a negative string test cannot rule out hvKP infection, and empirical anti-infective therapy against hypervirulent strains should not be withheld solely based on a negative string test result.

ST23 type was dominant (67.5%) in this research. Previous studies have shown that ST23 is a representative type of hvKP (Neumann et al., 2023). Capsular serotype was dominated by K1 (72.5%), followed by K2 (22.5%), and *wzi* typing showed that *wzi*1 was dominant (65%). This suggests that K1 and K2 are the most prevalent serotypes for EKE in this region, suggesting that K1 serotypes and wzi1 may be associated with higher pathogenicity, which may be due to the high virulence characteristics of K1 capsules (Feldman et al., 2019). Currently, CPS conjugate vaccines (such as K1 and K2 serotype vaccines) prepared by phage depolymerase have shown good protective effects in animal models (Ke et al., 2025), which can further promote clinical translational research in the future.

The phylogenetic tree of this research revealed that the strains in branches A-E all belong to the ST23-K1 type. Particularly, the strains in the E branch included those from liver abscesses in multiple regions of Asia, suggesting that the ST23-K1 type KP exists in a common genetic basis in East Asia and other regions (Jiang et al., 2025). Moreover, this clonal group also contained strains from liver abscesses and endophthalmitis. This indicates that when KP infections (such as liver abscesses and endophthalmitis) are encountered in clinical settings, especially in ophthalmology and infectious diseases departments, one should be highly vigilant of the possibility of ST23-K1 type hvKP. The strains in the F branch have more diverse molecular typing. The specimens came from blood, urine, sputum, abdominal abscesses, skin swabs, bronchoalveolar lavage fluid, etc., and were from multiple provinces in China and other countries (the United States, South Korea, Colombia, France, etc.). It is noteworthy that the ST11 type in this branch is often associated with the K64 serotype, and the ST11-K64 type is the recognized prevalent typing for carbapenem-resistant Klebsiella pneumoniae (CRKP) (Zhou et al., 2023; Budia-Silva et al., 2024). A part of the EKE strains in this research have a close relationship with the ST11-K64 strains in the public database, and the drug sensitivity results showed that one strain in this research was an ESBL-resistant strain. Therefore, in clinical treatment, even if the strain shows sensitivity to antibiotics, one should still be vigilant of its potential resistance background.

Due to the rapid progression of EnE, the initial visual acuity of most patients had dropped to the level of only remaining light perception at the time of their visit. In this research, 95.15% (98/103) of the patients had a poor visual prognosis in the end. Among them, 36 patients underwent enucleation of the eyeball. Low initial visual acuity is a risk factor for poor visual prognosis, which is consistent with the conclusion of Lee et al.’s study (Lee et al., 2022). At the same time, we found that intravitreal injection and immunosuppressive state can reduce the risk of poor prognosis. A total of 24 patients in this research received intravitreal injection. Intravitreal injection can enable antibacterial drugs to act directly on the lesion, which is more targeted and effective than intravenous administration. Therefore, it is an essential treatment method for EnE patients (VanDolah and Tung, 2023).

In this research, among the 37 patients who underwent vitrectomy, only 1 eventually required enucleation of the eye, while the remaining 36 cases (97.3%) successfully retained their eyes. Among the 36 patients who successfully retained their eyes, 34 had effective vision upon discharge, while 2 were blind. For the majority of patients, preserving the eye is not only about maintaining a slight vision, but also holds significant psychological and social importance. It helps to avoid the changes in image and psychological trauma caused by eye loss and also retains the hope for possible future visual rehabilitation treatment.

Although this research has explored the clinical characteristics, pathogenicology, and molecular epidemiology of EnE, it still has the following limitations: It was a single-center retrospective study with a limited sample size, and only partial strains were sequenced, while long-term follow-up data were unavailable for comprehensive prognostic assessment. Fungal pathogens were not systematically screened, which may lead to missed diagnoses. In addition, key information including antibiotic regimens and timing of intervention was incompletely recorded. Moreover, no control group was included in risk factor analysis, making it difficult to identify specific pathogenic factors for EKE. Future multi-center, prospective, large-sample cohort studies with long-term follow-up and molecular mechanism exploration will be conducted to address these shortcomings.

## 5 Conclusion

The incidence of EnE is increasing globally, with KP being the predominant pathogen. For diagnosis, patients presenting with only ocular symptoms should undergo routine systemic screening, including chest and abdominal imaging. Conversely, patients diagnosed with a liver abscess, particularly KP-related, should be actively assessed and monitored for ocular symptoms to detect potential EnE. Clinically, a negative string test cannot rule out infection caused by hvKP. Empirical anti-infective therapy targeting hypervirulent strains should not be withheld solely based on a negative string test result. When treating KP-related infections, vigilance for the hypervirulent ST23-K1 strain (hvKP) is crucial, and its potential for resistance should be considered even with susceptible *in vitro* results. Regarding treatment, initial low vision is a risk factor for poor visual outcome. Intravitreal injections can significantly improve prognosis and should be administered proactively. Vitrectomy also holds substantial value in infection control and globe preservation.

## Data Availability

The datasets presented in this study can be found in online repositories. The names of the repository/repositories and accession number(s) can be found in the **Supplementary Material**.
