# Supplementary material for: Retrospective single-center study of endogenous endophthalmitis, Northeastern China, 2010-2023
Source: Front Cell Infect Microbiol. 2026 Sep 3;16:1805346. doi: 10.3389/fcimb.2026.1805346 (PMC13581886; doi:10.3389/fcimb.2026.1805346)
Supplement: Supplementary file 1 [file DataSheet1.pdf]

Annexed Table1

Sample	HQ Reads	HQ Reads %	HQ Data (bp)	HQ Data %	Depth
A1	7,884,282	96.83	1,186,390,628	96.5	230
A10	9,736,556	97.16	1,464,740,946	96.8	259
A11	8,706,660	97.14	1,309,880,309	96.78	244
A12	8,034,580	96.75	1,208,551,542	96.37	219
A13	8,896,682	97.24	1,338,477,065	96.88	240
A14	9,468,404	96.97	1,424,333,558	96.6	247
A15	7,147,118	97.54	1,075,460,509	97.2	195
A16	8,050,952	97.46	1,211,614,876	97.14	215
A17	9,326,302	97.27	1,403,005,603	96.9	249
A18	8,012,868	96.97	1,205,132,472	96.58	214
A19	7,946,206	97.24	1,195,646,323	96.9	210
A2	8,287,884	96.85	1,246,937,957	96.5	236
A20	9,325,146	97.25	1,403,414,990	96.93	254
A21	9,163,616	97.51	1,378,914,982	97.18	247
A22	8,772,396	96.92	1,319,271,482	96.53	240
A23	8,222,758	96.83	1,237,155,256	96.48	221
A24	8,256,312	96.78	1,241,967,645	96.41	224
A25	8,211,202	97.09	1,235,488,197	96.75	222
A26	7,503,460	97.25	1,128,986,197	96.9	203
A27	8,652,200	97.23	1,301,741,283	96.87	240
A28	6,613,990	97.55	995,326,132	97.22	168
A29	7,217,488	97.18	1,086,049,951	96.84	195
A3	8,416,660	96.94	1,266,365,558	96.59	228
A30	7,392,614	97.19	1,112,235,651	96.84	197
A31	8,690,706	97.26	1,307,255,272	96.89	234
A32	7,921,376	97.29	1,191,734,025	96.93	213
A33	7,648,290	96.9	1,150,247,590	96.51	202
A34	8,723,548	96.9	1,311,941,377	96.51	241
A35	7,035,702	97.39	1,058,926,354	97.07	189
A36	8,865,874	96.97	1,333,759,974	96.61	242
A37	7,621,506	96.99	1,146,670,431	96.64	199
A38	7,465,474	97.13	1,122,949,324	96.75	198
A39	7,375,298	97.26	1,109,686,572	96.91	199

A4	8,966,362	97.67	1,349,824,307	97.37	235
A40	7,751,340	97.18	1,166,442,337	96.85	210
A5	9,841,778	97.34	1,480,709,964	96.98	269
A6	9,130,634	97.04	1,373,310,203	96.65	242
A7	8,953,274	97.41	1,347,524,631	97.09	250
A8	8,540,390	97.41	1,285,039,749	97.06	230
A9	8,613,616	97.36	1,296,079,750	97.02	232

Annexed Table2

Sample	Property	Contig	Scaffold
A1	Total sequence number	44	33
	Total sequence length	5,138,034	5,138,034
	Max sequence length	510,791	737,840
	Min sequence length	535	535
	N20	441,076	679,933
	N50	304,662	441,076
	N90	95,642	127,440
	N number	0	1,100
	N rate	0	0
	GC content %	58	58
	Sequences greater than 1kb	39	28
A10	Total sequence number	74	67
	Total sequence length	5,635,415	5,635,415
	Max sequence length	699,650	699,650
	Min sequence length	502	502
	N20	328,476	440,617
	N50	209,881	241,029
	N90	72,649	78,867
	N number	0	700
	N rate	0	0
	GC content %	57	57

	Sequences greater than 1kb	60	54
A11	Total sequence number	44	32
	Total sequence length	5,364,706	5,364,706
	Max sequence length	708,579	776,332
	Min sequence length	526	526
	N20	625,997	628,874
	N50	290,046	431,509
	N90	95,783	107,810
	N number	0	1,200
	N rate	0	0
	GC content %	57	57
	Sequences greater than 1kb	42	30
A12	Total sequence number	62	54
	Total sequence length	5,506,229	5,506,229
	Max sequence length	703,604	703,604
	Min sequence length	522	522
	N20	418,340	461,903
	N50	185,888	203,595
	N90	79,504	88,041
	N number	0	800
	N rate	0	0
	GC content %	57	57
	Sequences greater than 1kb	53	45
A13	Total sequence number	63	57
	Total sequence length	5,571,059	5,571,059
	Max sequence length	636,522	692,945
	Min sequence length	522	522
	N20	458,317	475,927
	N50	210,974	289,341

	N90	76,168	97,636
	N number	0	600
	N rate	0	0
	GC content %	57	57
	Sequences greater than 1kb	53	47
A14	Total sequence number	81	72
	Total sequence length	5,758,432	5,758,432
	Max sequence length	704,505	704,505
	Min sequence length	522	522
	N20	381,021	413,837
	N50	209,873	283,213
	N90	62,125	91,649
	N number	0	900
	N rate	0	0
	GC content %	57	57
	Sequences greater than 1kb	69	60
A15	Total sequence number	54	48
	Total sequence length	5,501,709	5,501,709
	Max sequence length	704,583	843,748
	Min sequence length	522	522
	N20	667,457	704,583
	N50	209,879	326,442
	N90	80,522	86,397
	N number	0	600
	N rate	0	0
	GC content %	57	57
	Sequences greater than 1kb	46	40
A16	Total sequence number	63	54
	Total sequence length	5,622,784	5,622,784

	Max sequence length	704,439	704,439
	Min sequence length	522	522
	N20	481,078	481,078
	N50	217,207	289,293
	N90	72,878	91,650
	N number	0	900
	N rate	0	0
	GC content %	57	57
	Sequences greater than 1kb	55	46
A17	Total sequence number	75	68
	Total sequence length	5,634,025	5,634,025
	Max sequence length	488,243	488,243
	Min sequence length	190	522
	N20	326,863	326,863
	N50	172,649	194,173
	N90	62,965	72,841
	N number	0	700
	N rate	0	0
	GC content %	57	57
	Sequences greater than 1kb	63	57
A18	Total sequence number	63	55
	Total sequence length	5,611,725	5,611,725
	Max sequence length	696,757	696,757
	Min sequence length	522	522
	N20	475,898	480,897
	N50	233,280	289,302
	N90	79,321	99,379
	N number	0	800
	N rate	0	0

	GC content %	57	57
	Sequences greater than 1kb	53	45
A19	Total sequence number	70	62
	Total sequence length	5,688,702	5,688,702
	Max sequence length	458,054	672,586
	Min sequence length	190	502
	N20	326,861	402,420
	N50	214,432	264,872
	N90	72,878	86,398
	N number	0	800
	N rate	0	0
	GC content %	57	57
	Sequences greater than 1kb	57	50
A2	Total sequence number	41	32
	Total sequence length	5,283,596	5,283,596
	Max sequence length	599,094	599,094
	Min sequence length	205	526
	N20	475,108	475,108
	N50	294,435	368,115
	N90	92,898	130,112
	N number	0	900
	N rate	0	0
	GC content %	57	57
	Sequences greater than 1kb	38	30
A20	Total sequence number	64	53
	Total sequence length	5,509,687	5,509,687
	Max sequence length	507,649	533,338
	Min sequence length	190	584
	N20	312,477	427,834

	N50	196,909	232,159
	N90	83,717	98,371
	N number	0	1,100
	N rate	0	0
	GC content %	57	57
	Sequences greater than 1kb	59	49
A21	Total sequence number	53	49
	Total sequence length	5,569,740	5,569,740
	Max sequence length	646,830	703,429
	Min sequence length	522	522
	N20	414,406	629,060
	N50	288,988	326,861
	N90	72,877	86,397
	N number	0	400
	N rate	0	0
	GC content %	57	57
	Sequences greater than 1kb	45	41
A22	Total sequence number	63	54
	Total sequence length	5,485,296	5,485,296
	Max sequence length	734,220	864,169
	Min sequence length	522	522
	N20	436,521	436,521
	N50	201,817	244,417
	N90	72,893	94,357
	N number	0	900
	N rate	0	0
	GC content %	57	57
	Sequences greater than 1kb	57	48
A23	Total sequence number	92	86

	Total sequence length	5,596,741	5,596,741
	Max sequence length	933,499	933,499
	Min sequence length	121	502
	N20	328,474	440,615
	N50	186,469	203,595
	N90	46,845	49,003
	N number	0	600
	N rate	0	0
	GC content %	57	57
	Sequences greater than 1kb	72	67
A24	Total sequence number	52	46
	Total sequence length	5,522,634	5,522,634
	Max sequence length	704,435	704,435
	Min sequence length	522	522
	N20	467,560	670,649
	N50	313,600	326,861
	N90	86,397	91,650
	N number	0	600
	N rate	0	0
	GC content %	57	57
	Sequences greater than 1kb	45	39
A25	Total sequence number	59	47
	Total sequence length	5,553,056	5,553,056
	Max sequence length	633,026	1,226,440
	Min sequence length	537	537
	N20	603,002	1,226,440
	N50	308,500	376,632
	N90	86,427	99,985
	N number	0	1,200

	N rate	0	0
	GC content %	57	57
	Sequences greater than 1kb	56	44
A26	Total sequence number	53	46
	Total sequence length	5,558,742	5,558,742
	Max sequence length	648,085	704,508
	Min sequence length	522	522
	N20	467,637	670,650
	N50	210,954	306,693
	N90	79,363	99,379
	N number	0	700
	N rate	0	0
	GC content %	57	57
	Sequences greater than 1kb	45	38
A27	Total sequence number	55	46
	Total sequence length	5,412,564	5,412,564
	Max sequence length	518,827	518,827
	Min sequence length	312	718
	N20	383,929	428,441
	N50	212,500	303,532
	N90	79,265	86,388
	N number	0	900
	N rate	0	0
	GC content %	57	57
	Sequences greater than 1kb	53	45
A28	Total sequence number	90	76
	Total sequence length	5,923,887	5,923,887
	Max sequence length	719,940	719,940
	Min sequence length	530	530

	N20	414,426	617,106
	N50	185,564	320,737
	N90	81,230	86,443
	N number	0	1,400
	N rate	0	0
	GC content %	57	57
	Sequences greater than 1kb	69	55
A29	Total sequence number	70	58
	Total sequence length	5,568,558	5,568,558
	Max sequence length	427,328	534,005
	Min sequence length	584	584
	N20	303,548	428,056
	N50	185,268	212,592
	N90	66,793	81,863
	N number	0	1,200
	N rate	0	0
	GC content %	57	57
	Sequences greater than 1kb	65	53
A3	Total sequence number	81	75
	Total sequence length	5,538,251	5,538,251
	Max sequence length	570,792	627,391
	Min sequence length	190	502
	N20	306,684	306,684
	N50	182,782	185,888
	N90	56,499	59,956
	N number	0	600
	N rate	0	0
	GC content %	57	57
	Sequences greater than 1kb	69	64

A30	Total sequence number	69	60
	Total sequence length	5,625,878	5,625,878
	Max sequence length	650,828	650,828
	Min sequence length	522	522
	N20	475,916	475,916
	N50	209,881	289,302
	N90	59,605	86,397
	N number	0	900
	N rate	0	0
	GC content %	57	57
	Sequences greater than 1kb	60	51
A31	Total sequence number	79	70
	Total sequence length	5,579,572	5,579,572
	Max sequence length	566,438	566,438
	Min sequence length	94	554
	N20	560,560	560,560
	N50	196,204	301,398
	N90	52,005	59,575
	N number	0	900
	N rate	0	0
	GC content %	57	57
	Sequences greater than 1kb	72	65
A32	Total sequence number	66	58
	Total sequence length	5,594,107	5,594,107
	Max sequence length	647,507	703,930
	Min sequence length	190	522
	N20	481,074	481,074
	N50	210,968	289,341
	N90	76,036	91,650

	N number	0	800
	N rate	0	0
	GC content %	57	57
	Sequences greater than 1kb	57	50
A33	Total sequence number	71	59
	Total sequence length	5,693,187	5,693,187
	Max sequence length	515,949	518,825
	Min sequence length	94	552
	N20	229,815	374,306
	N50	155,328	225,411
	N90	79,424	99,393
	N number	0	1,200
	N rate	0	0
	GC content %	57	57
	Sequences greater than 1kb	62	51
A34	Total sequence number	67	61
	Total sequence length	5,427,934	5,427,934
	Max sequence length	629,854	917,898
	Min sequence length	522	522
	N20	326,642	381,008
	N50	203,587	203,587
	N90	59,586	85,834
	N number	0	600
	N rate	0	0
	GC content %	57	57
	Sequences greater than 1kb	56	50
A35	Total sequence number	79	72
	Total sequence length	5,580,466	5,580,466
	Max sequence length	481,379	537,978

	Min sequence length	502	502
	N20	306,675	408,967
	N50	194,140	210,968
	N90	68,574	68,973
	N number	0	700
	N rate	0	0
	GC content %	57	57
	Sequences greater than 1kb	66	59
A36	Total sequence number	69	59
	Total sequence length	5,504,381	5,504,381
	Max sequence length	502,478	507,247
	Min sequence length	606	606
	N20	303,690	360,931
	N50	196,966	212,349
	N90	70,251	86,388
	N number	0	1,000
	N rate	0	0
	GC content %	57	57
	Sequences greater than 1kb	65	55
A37	Total sequence number	75	64
	Total sequence length	5,751,684	5,751,684
	Max sequence length	399,338	704,702
	Min sequence length	522	522
	N20	313,692	335,568
	N50	200,109	283,739
	N90	63,904	76,275
	N number	0	1,100
	N rate	0	0
	GC content %	57	57

	Sequences greater than 1kb	65	54
A38	Total sequence number	64	60
	Total sequence length	5,663,614	5,663,614
	Max sequence length	694,513	694,513
	Min sequence length	522	522
	N20	481,089	481,089
	N50	233,280	289,345
	N90	76,828	91,650
	N number	0	400
	N rate	0	0
	GC content %	57	57
	Sequences greater than 1kb	52	48
A39	Total sequence number	70	63
	Total sequence length	5,554,253	5,554,253
	Max sequence length	703,026	703,026
	Min sequence length	522	522
	N20	332,788	335,664
	N50	203,592	262,662
	N90	67,188	86,397
	N number	0	700
	N rate	0	0
	GC content %	57	57
	Sequences greater than 1kb	58	51
A4	Total sequence number	67	60
	Total sequence length	5,734,736	5,734,736
	Max sequence length	703,981	703,981
	Min sequence length	190	522
	N20	475,913	480,805
	N50	211,010	289,356

	N90	79,504	91,650
	N number	0	700
	N rate	0	0
	GC content %	57	57
	Sequences greater than 1kb	54	48
A40	Total sequence number	59	53
	Total sequence length	5,540,803	5,540,803
	Max sequence length	530,710	745,662
	Min sequence length	312	584
	N20	343,875	507,292
	N50	212,410	218,198
	N90	98,226	99,058
	N number	0	600
	N rate	0	0
	GC content %	57	57
	Sequences greater than 1kb	55	50
A5	Total sequence number	75	69
	Total sequence length	5,498,506	5,498,506
	Max sequence length	369,904	369,904
	Min sequence length	190	522
	N20	326,861	326,861
	N50	201,875	261,059
	N90	59,577	66,937
	N number	0	600
	N rate	0	0
	GC content %	57	57
	Sequences greater than 1kb	64	59
A6	Total sequence number	67	61
	Total sequence length	5,668,250	5,668,250

	Max sequence length	938,130	938,130
	Min sequence length	522	522
	N20	440,982	440,982
	N50	223,028	289,367
	N90	64,100	67,091
	N number	0	600
	N rate	0	0
	GC content %	57	57
	Sequences greater than 1kb	55	50
A7	Total sequence number	49	39
	Total sequence length	5,375,545	5,375,545
	Max sequence length	512,231	738,213
	Min sequence length	535	535
	N20	441,077	679,837
	N50	304,749	441,077
	N90	86,378	127,932
	N number	0	1,000
	N rate	0	0
	GC content %	57	57
	Sequences greater than 1kb	41	31
A8	Total sequence number	70	61
	Total sequence length	5,575,017	5,575,017
	Max sequence length	644,781	701,380
	Min sequence length	522	522
	N20	470,669	470,669
	N50	203,595	262,661
	N90	72,878	86,397
	N number	0	900
	N rate	0	0

	GC content %	57	57
	Sequences greater than 1kb	58	49
A9	Total sequence number	86	76
	Total sequence length	5,586,453	5,586,453
	Max sequence length	431,294	487,717
	Min sequence length	190	522
	N20	306,684	326,849
	N50	172,607	194,617
	N90	53,446	68,304
	N number	0	1,000
	N rate	0	0
	GC content %	57	57
	Sequences greater than 1kb	73	64

Annexed table3

Years	Endogenous endophthalmitis	Exogenous endophthalmitis	Total	The proportion of EnE
2010	0	9	9	0. 00%
2011	3	35	38	7. 89%
2012	3	21	24	12. 50%
2013	7	17	24	29. 17%
2014	2	13	15	13. 33%
2015	6	12	18	33. 33%
2016	6	13	19	31. 58%
2017	9	15	24	37. 50%
2018	6	11	17	35. 29%
2019	13	15	28	46. 43%
2020	12	15	27	44. 44%
2021	20	19	39	51. 28%
2022	5	11	16	31. 25%
2023	11	11	22	50. 00%
