# Supplementary material for: Retrospective single-center study of endogenous endophthalmitis, Northeastern China, 2010-2023
Source: Front Cell Infect Microbiol. 2026 Sep 3;16:1805346. doi: 10.3389/fcimb.2026.1805346 (PMC13581886; doi:10.3389/fcimb.2026.1805346)
Supplement: Supplementary file 2 [file SupplementaryFile1.docx]

Accession	Sample Name	SPUID	Organism	Tax ID	Strain	BioProject

SAMN59993439	KP_Z	KP_Z	Klebsiella pneumoniae	573	KP_Z	PRJNA1465681

Accession    Sample Name    SPUID    Organism    Tax ID    Strain    BioProject
[https://www.ncbi.nlm.nih.gov/biosample/59993439](https://www.ncbi.nlm.nih.gov/biosample/59993439)
