# Supplementary material for: Retrospective single-center study of endogenous endophthalmitis, Northeastern China, 2010-2023
Source: Front Cell Infect Microbiol. 2026 Sep 3;16:1805346. doi: 10.3389/fcimb.2026.1805346 (PMC13581886; doi:10.3389/fcimb.2026.1805346)
Supplement: Supplementary file 3 [file SupplementaryFile2.docx]

40KP

Object IDs and corresponding URLs:

59975230: <https://www.ncbi.nlm.nih.gov/wgs_batch/59975230>

59975231: https://www.ncbi.nlm.nih.gov/wgs_batch/59975231

59975232: https://www.ncbi.nlm.nih.gov/wgs_batch/59975232

59975233: https://www.ncbi.nlm.nih.gov/wgs_batch/59975233

59975234: https://www.ncbi.nlm.nih.gov/wgs_batch/59975234

59975235: https://www.ncbi.nlm.nih.gov/wgs_batch/59975235

59975236: https://www.ncbi.nlm.nih.gov/wgs_batch/59975236

59975237: https://www.ncbi.nlm.nih.gov/wgs_batch/59975237

59975238: https://www.ncbi.nlm.nih.gov/wgs_batch/59975238

59975239: https://www.ncbi.nlm.nih.gov/wgs_batch/59975239

59975240: https://www.ncbi.nlm.nih.gov/wgs_batch/59975240

59975241: https://www.ncbi.nlm.nih.gov/wgs_batch/59975241

59975242: https://www.ncbi.nlm.nih.gov/wgs_batch/59975242

59975243: https://www.ncbi.nlm.nih.gov/wgs_batch/59975243

59975244: https://www.ncbi.nlm.nih.gov/wgs_batch/59975244

59975245: https://www.ncbi.nlm.nih.gov/wgs_batch/59975245

59975246: https://www.ncbi.nlm.nih.gov/wgs_batch/59975246

59975247: https://www.ncbi.nlm.nih.gov/wgs_batch/59975247

59975248: https://www.ncbi.nlm.nih.gov/wgs_batch/59975248

59975249: https://www.ncbi.nlm.nih.gov/wgs_batch/59975249

59975250: https://www.ncbi.nlm.nih.gov/wgs_batch/59975250

59975251: https://www.ncbi.nlm.nih.gov/wgs_batch/59975251

59975252: https://www.ncbi.nlm.nih.gov/wgs_batch/59975252

59975253: https://www.ncbi.nlm.nih.gov/wgs_batch/59975253

59975254: https://www.ncbi.nlm.nih.gov/wgs_batch/59975254

59975255: https://www.ncbi.nlm.nih.gov/wgs_batch/59975255

59975256: https://www.ncbi.nlm.nih.gov/wgs_batch/59975256

59975257: https://www.ncbi.nlm.nih.gov/wgs_batch/59975257

59975258: https://www.ncbi.nlm.nih.gov/wgs_batch/59975258

59975259: https://www.ncbi.nlm.nih.gov/wgs_batch/59975259

59975260: https://www.ncbi.nlm.nih.gov/wgs_batch/59975260

59975261: https://www.ncbi.nlm.nih.gov/wgs_batch/59975261

59975262: https://www.ncbi.nlm.nih.gov/wgs_batch/59975262

59975263: https://www.ncbi.nlm.nih.gov/wgs_batch/59975263

59975264: https://www.ncbi.nlm.nih.gov/wgs_batch/59975264

59975265: https://www.ncbi.nlm.nih.gov/wgs_batch/59975265

59975266: https://www.ncbi.nlm.nih.gov/wgs_batch/59975266

59975267: https://www.ncbi.nlm.nih.gov/wgs_batch/59975267

59975268: https://www.ncbi.nlm.nih.gov/wgs_batch/59975268

59975269: https://www.ncbi.nlm.nih.gov/wgs_batch/59975269

59975230: https://www.ncbi.nlm.nih.gov/wgs_batch/59975230

59975231: https://www.ncbi.nlm.nih.gov/wgs_batch/59975231

59975232: https://www.ncbi.nlm.nih.gov/wgs_batch/59975232

59975233: https://www.ncbi.nlm.nih.gov/wgs_batch/59975233

59975234: https://www.ncbi.nlm.nih.gov/wgs_batch/59975234

59975235: https://www.ncbi.nlm.nih.gov/wgs_batch/59975235

59975236: https://www.ncbi.nlm.nih.gov/wgs_batch/59975236

59975237: https://www.ncbi.nlm.nih.gov/wgs_batch/59975237

59975238: https://www.ncbi.nlm.nih.gov/wgs_batch/59975238

59975239: https://www.ncbi.nlm.nih.gov/wgs_batch/59975239

59975240: https://www.ncbi.nlm.nih.gov/wgs_batch/59975240

59975241: https://www.ncbi.nlm.nih.gov/wgs_batch/59975241

59975242: https://www.ncbi.nlm.nih.gov/wgs_batch/59975242

59975243: https://www.ncbi.nlm.nih.gov/wgs_batch/59975243

59975244: https://www.ncbi.nlm.nih.gov/wgs_batch/59975244

59975245: https://www.ncbi.nlm.nih.gov/wgs_batch/59975245

59975246: https://www.ncbi.nlm.nih.gov/wgs_batch/59975246

59975247: https://www.ncbi.nlm.nih.gov/wgs_batch/59975247

59975248: https://www.ncbi.nlm.nih.gov/wgs_batch/59975248

59975249: https://www.ncbi.nlm.nih.gov/wgs_batch/59975249

59975250: https://www.ncbi.nlm.nih.gov/wgs_batch/59975250

59975251: https://www.ncbi.nlm.nih.gov/wgs_batch/59975251

59975252: https://www.ncbi.nlm.nih.gov/wgs_batch/59975252

59975253: https://www.ncbi.nlm.nih.gov/wgs_batch/59975253

59975254: https://www.ncbi.nlm.nih.gov/wgs_batch/59975254

59975255: https://www.ncbi.nlm.nih.gov/wgs_batch/59975255

59975256: https://www.ncbi.nlm.nih.gov/wgs_batch/59975256

59975257: https://www.ncbi.nlm.nih.gov/wgs_batch/59975257

59975258: https://www.ncbi.nlm.nih.gov/wgs_batch/59975258

59975259: https://www.ncbi.nlm.nih.gov/wgs_batch/59975259

59975260: https://www.ncbi.nlm.nih.gov/wgs_batch/59975260

59975261: https://www.ncbi.nlm.nih.gov/wgs_batch/59975261

59975262: https://www.ncbi.nlm.nih.gov/wgs_batch/59975262

59975263: https://www.ncbi.nlm.nih.gov/wgs_batch/59975263

59975264: https://www.ncbi.nlm.nih.gov/wgs_batch/59975264

59975265: https://www.ncbi.nlm.nih.gov/wgs_batch/59975265

59975266: https://www.ncbi.nlm.nih.gov/wgs_batch/59975266

59975267: https://www.ncbi.nlm.nih.gov/wgs_batch/59975267

59975268: https://www.ncbi.nlm.nih.gov/wgs_batch/59975268

59975269: <https://www.ncbi.nlm.nih.gov/wgs_batch/59975269>

Accession	Sample Name	SPUID	Organism	Tax ID	Strain	BioProject

SAMN59975230	A1	A1	Klebsiella pneumoniae	573	A1	PRJNA1465681

SAMN59975231	A2	A2	Klebsiella pneumoniae	573	A2	PRJNA1465681

SAMN59975232	A3	A3	Klebsiella pneumoniae	573	A3	PRJNA1465681

SAMN59975233	A4	A4	Klebsiella pneumoniae	573	A4	PRJNA1465681

SAMN59975234	A5	A5	Klebsiella pneumoniae	573	A5	PRJNA1465681

SAMN59975235	A6	A6	Klebsiella pneumoniae	573	A6	PRJNA1465681

SAMN59975236	A7	A7	Klebsiella pneumoniae	573	A7	PRJNA1465681

SAMN59975237	A8	A8	Klebsiella pneumoniae	573	A8	PRJNA1465681

SAMN59975238	A9	A9	Klebsiella pneumoniae	573	A9	PRJNA1465681

SAMN59975239	A10	A10	Klebsiella pneumoniae	573	A10	PRJNA1465681

SAMN59975240	A11	A11	Klebsiella pneumoniae	573	A11	PRJNA1465681

SAMN59975241	A12	A12	Klebsiella pneumoniae	573	A12	PRJNA1465681

SAMN59975242	A13	A13	Klebsiella pneumoniae	573	A13	PRJNA1465681

SAMN59975243	A14	A14	Klebsiella pneumoniae	573	A14	PRJNA1465681

SAMN59975244	A15	A15	Klebsiella pneumoniae	573	A15	PRJNA1465681

SAMN59975245	A16	A16	Klebsiella pneumoniae	573	A16	PRJNA1465681

SAMN59975246	A17	A17	Klebsiella pneumoniae	573	A17	PRJNA1465681

SAMN59975247	A18	A18	Klebsiella pneumoniae	573	A18	PRJNA1465681

SAMN59975248	A19	A19	Klebsiella pneumoniae	573	A19	PRJNA1465681

SAMN59975249	A20	A20	Klebsiella pneumoniae	573	A20	PRJNA1465681

SAMN59975250	A21	A21	Klebsiella pneumoniae	573	A21	PRJNA1465681

SAMN59975251	A22	A22	Klebsiella pneumoniae	573	A22	PRJNA1465681

SAMN59975252	A23	A23	Klebsiella pneumoniae	573	A23	PRJNA1465681

SAMN59975253	A24	A24	Klebsiella pneumoniae	573	A24	PRJNA1465681

SAMN59975254	A25	A25	Klebsiella pneumoniae	573	A25	PRJNA1465681

SAMN59975255	A26	A26	Klebsiella pneumoniae	573	A26	PRJNA1465681

SAMN59975256	A27	A27	Klebsiella pneumoniae	573	A27	PRJNA1465681

SAMN59975257	A28	A28	Klebsiella pneumoniae	573	A28	PRJNA1465681

SAMN59975258	A29	A29	Klebsiella pneumoniae	573	A29	PRJNA1465681

SAMN59975259	A30	A30	Klebsiella pneumoniae	573	A30	PRJNA1465681

SAMN59975260	A31	A31	Klebsiella pneumoniae	573	A31	PRJNA1465681

SAMN59975261	A32	A32	Klebsiella pneumoniae	573	A32	PRJNA1465681

SAMN59975262	A33	A33	Klebsiella pneumoniae	573	A33	PRJNA1465681

SAMN59975263	A34	A34	Klebsiella pneumoniae	573	A34	PRJNA1465681

SAMN59975264	A35	A35	Klebsiella pneumoniae	573	A35	PRJNA1465681

SAMN59975265	A36	A36	Klebsiella pneumoniae	573	A36	PRJNA1465681

SAMN59975266	A37	A37	Klebsiella pneumoniae	573	A37	PRJNA1465681

SAMN59975267	A38	A38	Klebsiella pneumoniae	573	A38	PRJNA1465681

SAMN59975268	A39	A39	Klebsiella pneumoniae	573	A39	PRJNA1465681

SAMN59975269	A40	A40	Klebsiella pneumoniae	573	A40	PRJNA1465681
